# Early and late fixation of ulnar styloid base fractures yields different outcomes

**DOI:** 10.1186/s13018-018-0899-6

**Published:** 2018-07-31

**Authors:** Alvin Chao-Yu Chen, Chih-Hao Chiu, Chun-Jui Weng, Shih-Sheng Chang, Chun-Ying Cheng

**Affiliations:** Bone and Joint Research Center, Department of Orthopaedic Surgery, Chang Gung Memorial Hospital–Linkou and Chang Gung University College of Medicine, 5th, Fu-Shin Street, Kweishan District, Taoyuan, 333 Taiwan, Republic of China

**Keywords:** Ulnar styloid, Distal radioulnar joint (DRUJ), Triangular fibrocartilage complex (TFCC), Distal radius fracture (DRF)

## Abstract

**Background:**

The role of surgical fixation of ulnar styloid fractures remains a subject of debate. The purpose of this study was to compare the surgical outcomes following early and late intervention.

**Methods:**

We retrospectively reviewed 28 patients who underwent surgical repair for unilateral ulnar styloid fractures with distal radioulnar instability between 2004 and 2014. Surgical fixation was performed within 3 months of injury in 13 patients (group A) and beyond 3 months in 15 patients (group B). Patient characteristics and functional outcomes were compared between the two groups. The outcome survey consisted of QuickDASH score, grip strength, range of motion, pain score based on the visual analog scale, and surgical complications. Descriptive statistics were calculated for key variables. A *p* value of < 0.01 was considered statistically significant.

**Results:**

Patient characteristics including age, sex, injured side, dominant side injury, and concomitant distal radius fracture showed no significant differences between the two groups. Time to surgery averaged 1.1 months in group A and 12.3 months in group B. Significantly better outcomes were found in group A than in group B, including QuickDASH scores (4.4 ± 5.9 vs. 12.9 ± 9.9) and grip strength (37.4 ± 5.1 vs. 29.1 ± 5.9 kg). Significantly better range of motion was found in group A than in group B with respect to supination (81.9° ± 4.3° vs. 75° ± 8.5°), extension (84.6 ± 4.3 vs. 76.7 ± 6.5), and flexion (80.4° ± 3.8° vs. 72° ± 4.1°). The difference was not significant in case of pronation (78.8° ± 3° vs. 74.3° ± 5.9°) and with respect to pain scores (0.6 ± 0.7 vs. 1.3 ± 1).

**Conclusion:**

Both osseous and soft tissue lesions need to be fully addressed in ulnar styloid fractures. Early detection and surgical repair yielded better outcomes. Higher complication rates in late-treated fractures show that surgeons should select surgical candidates and modalities properly.

## Background

An ulnar styloid fracture is common and may be associated with a distal radial fracture (DRF) or occurs as an isolated injury [1]. In spite of acting as a unique strut on the ulnar end to stabilize the ulnar soft tissue and maintain the congruency of the distal radioulnar joint (DRUJ), the majority of these connected tissues including the triangular fibrocartilage complex (TFCC) are at the base of the ulnar styloid or fovea [2–4]. These tissue attachments generally allow two morphologically and functionally different fracture types with ulnar styloid injury. One type is a fracture of the ulnar styloid tip, in which the TFCC remains intact; the other type is a styloid base fracture, which is the result of an avulsion of the ulnar TFCC attachment [5]. Anatomical relationships and biomechanical implications have been confirmed for ulnar styloid and DRUJ stabilizers [6]. Given the rising concern about the presence of an ulnar styloid with residual ulnocarpal complains [7], current studies still questioned the potential advantages of surgical fixation of these fractures [8–10]. Updated reports regarding sequelae of untreated distal ulnar fractures were either confined to cadaver studies [11] or had a small sample size [12]. The purpose of this study was to discuss the presentation in a case series of ulnar styloid fractures and to compare the treatment outcomes following early surgical intervention or late management.

## Methods

### Patient data

Ethics committee approval was obtained from the Chang Gung Institutional Review Board (IRB 201800834B0). A total of 37 patients were identified from our surgical database who underwent surgical fixation for unilateral ulnar styloid fracture between 2004 and 2014. All were displaced fractures with concomitant DRUJ instability. Four patients with concomitant carpal bone fracture dislocation and five patients lost to follow-up including two expired due to unrelated causes and three moving abroad were excluded. A total of 28 patients were enrolled in this retrospective study. There were 17 male and 11 female patients with an average age of 32.4 ± 12.9 years (range, 17 to 60 years). The time from injury to index surgery averaged 7.1 ± 7.7 months, range 0 (within 3 days) to 24 months. The patients were divided into two groups based on time interval from injury to fracture fixation. Group A included patients who underwent surgical fixation within 3 months of injury, with group B, beyond 3 months after injury. There were 13 patients in group A and 15 in group B. The average age was 36.1 ± 14.2 years (range, 18 to 60) in group A and 29.3 ± 11.2 years (range, 17 to 55) in group B. Characteristics of both groups are summarized in Table 1.

**Table 1:** Demographic data

Characteristics	Group A^†^	Group B^‡^	*p* value
No. of patients	13	15	
Mean age (years)	36.1 ± 14.2	29.3 ± 11.2	0.084
Sex			0.196
Women	6 (46%)	5 (33%)	
Men	7 (54%)	10 (67%)	
Injured wrist			0.147
Right	6 (46%)	10 (67%)	
Left	7 (54%)	5 (33%)	
Dominant side injury	6 (46%)	8 (53%)	0.359
Concomitant DRF	3	2	0.26
Time to surgery (months)	1.1	12.3	*0.000*

*p* values in italics represent statistical significance (*p* < 0.01)

*DRF* distal radial fracture

^†^Patients who underwent surgery within 3 months of injury

^‡^Patients who underwent surgery 3 months or more after injury

### Surgical procedure

**Fig. 1:**
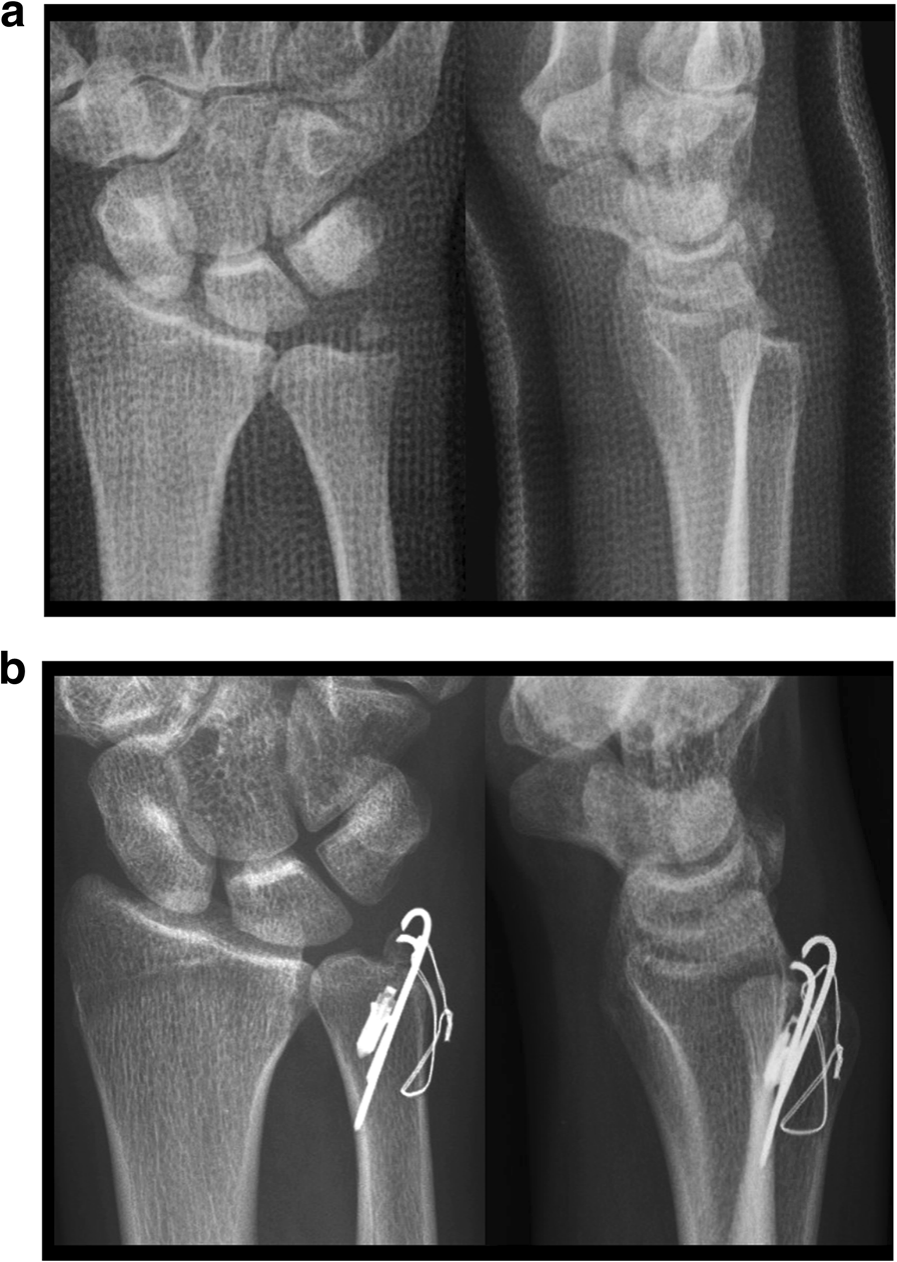
Twenty-one-year-old male. **a** Displaced ulnar styloid base fracture of the right wrist. **b** Fixation with Kirschner wires and steel ligature plus Mitek bone anchor

All surgeries were performed by a single surgeon. The patient was positioned supine with the elbow flexed and the forearm pronated. A 4- to 5-cm longitudinal incision was made over the dorso-ulnar side of the distal ulna, starting at the tip of the ulnar styloid and extending proximally. The dorsal sensory branch of the ulnar nerve (DSBUN) was identified and protected. The dorsal retinaculum was incised and the distal ulna was exposed directly through the interval between the flexor carpi ulnaris and extensor carpi ulnaris tendons. In acute injuries, the styloid fracture was easily identified. Gentle dissection was performed to preserve surrounding periosteal tissue, while the integrity of superficial and deep limbs of the TFCC was carefully checked at the insertion site. For chronic cases, a 5-mm osteotome was used to dissect the nonunion or malunion site. Decortication was meticulously performed to facilitate subsequent osteosynthesis. In case of deep TFCC fiber tear, a 2.0-mm Mitek bone anchor (Mitek Surgical Products, Norwood, MA, USA) was inserted slightly medial to the fracture site with suture passing the torn deep fiber while left untightened before fixation of the ulnar styloid. The ulnar styloid was reduced back to the distal ulna. Two 1.0-mm Kirschner wires were inserted from the styloid tip in a retrograde direction through the fracture site to securely purchase the opposite cortex of the distal ulna. A mini C arm image intensifier was used to confirm the fracture reduction as well as the DRUJ congruence. Double-loaded stainless-steel ligature wires were applied to serve as a tension band by looping the Kirschner wires distally and then passing through the cortex proximally to the fracture site. Optimal tension was applied by twisting the ligature wires appropriately, and then, the anchor suture was tightened (Fig. 1). With completion of styloid osteosynthesis and TFCC repair, the retinaculum and skin were closed with subcuticular sutures. After surgery, the wrist was protected in a short arm splint with the forearm and wrist in neutral rotation for 4 weeks. Gentle motion was started and return to work was allowed after 3 months postoperatively.

### Functional and radiographic survey

Postoperative visits were scheduled at out-patient clinics. Postoperative radiographs were taken the next day, at 3 months, and at the latest follow-up. Functional outcome measured at 2 years after surgery included range of motion of the wrist and forearm, grip strength, the Quick Disabilities of the Arm, Shoulder, and Hand (QuickDASH) score, and residual wrist pain. As a shortened version of the DASH outcome measure, the QuickDASH consists of 11 items (scored 1–5), instead of 30 items as in the DASH questionnaire, to evaluate perceived physical function and symptoms in individuals with upper limb musculoskeletal disorders [13].

Pain score was rated based on a 10-point visual analog scale (VAS). Osseous union was documented according to both clinical and radiographic assessment. Nonunion, malunion, infection, or complications associated with the implant, such as pain or tendonitis, were also based on the review of medical records.

### Statistical analysis

Descriptive statistics were calculated for key variables. For normally distributed data (patient age and time between fracture and fixation), an independent sample *t* test was used. For data that were not normally distributed (the QuickDASH score), the Mann-Whitney rank sum test was used. For categorical data (sex, injured side, injured hand dominance, concomitant DRF, and complication rate), a chi-square test was used. A *p* value of < 0.01 was considered statistically significant.

## Results

### Surgical data

All 28 patients had displaced ulnar styloid fractures with either instability or subluxation of the DRUJ. There were 2 revision cases (1 in each group) that failed prior DRUJ pinning. In group B, there were 13 patients with styloid nonunion and 2 with malunion. Four patients sustained concomitant DRF (3 in group A and 1 in group B) and 1 patient had a distal radial shaft fracture (Galeazzi injury) in group B. All 5 radial fractures underwent open reduction with locking plate fixation before ulnar styloid fixation. In addition to tension band wire fixation, suture anchors were applied for augmented TFCC repair in 10 patients (4 in group A and 6 in group B).

### Clinical outcome

Osseous union was confirmed both clinically and radiographically at 3 months after surgery in 25 patients (89%). Functional outcomes measured and compared between two groups at 2 years after surgery included QuickDASH scores, grip strength, range of motion, and pain scores (Table 2). The QuickDASH score averaged 8.9 ± 9.2 (range, 0 to 34.1) in all 28 patients, 4.4 ± 5.9 (range, 0 to 20.5) in group A and 12.9 ± 9.9 (range, 0 to 34.1) in group B, with a significant difference (*p* = 0.009). Grip strength averaged 33.2 ± 7.3 kg (range, 21 to 45), 37.4 ± 5.1 kg (range, 28 to 45) in group A and 29.1 ± 5.9 kg (range, 25 to 41) in group B, with a significant difference (*p* = 0.000). Range of motion was measured for four items including supination, pronation, extension, and flexion. Supination averaged 70° ± 13.2° (range, 55 to 85), 81.9° ± 4.3° (range, 70 to 85) in group A and 75° ± 8.5° (range, 55 to 85) in group B, with a significant difference (*p* = 0.007). Pronation averaged 75° ± 8.7°° (range, 65 to 85), 78.8° ± 3° (range 75 to 85) in group A and 74.3° ± 5.9° (range, 60 to 80) in group B. The difference was insignificant (*p* = 0.018). Extension averaged 80.3° ± 6.8° (range, 65 to 90), 84.6° ± 4.3° (range, 75 to 90) in group A and 76.7° ± 6.5° (range, 65 to 85) in group B, with a significant difference (*p* = 0.000). Flexion averaged 75.9° ± 5.8° (range, 60 to 85), 80.4° ± 3.8° (range, 75 to 85) in group A and 72° ± 4.1° (range, 60 to 75) in group B, with a significant difference (*p* = 0.000). The VAS pain score averaged 1 ± 0.9 (range, 0–3), 0.6 ± 0.7 (range, 0–2) in group A and 1.3 ± 1 (range, 0–3) in group B. The difference was insignificant (*p* = 0.025).

**Table 2:** Functional outcome

Items	Group A	Group B	*p* value
QuickDASH	4.4 ± 5.9	12.9 ± 9.9	*0.009*
Grip strength (kg)	37.4 ± 5.1	29.1 ± 5.9	*0.000*
Range of motion (degrees)			
Supination	81.9 ± 4.3	75 ± 8.5	*0.007*
Pronation	78.8 ± 3	74.3 ± 5.9	0.018
Extension	84.6 ± 4.3	76.7 ± 6.5	*0.000*
Flexion	80.4 ± 3.8	72 ± 4.1	*0.000*
Pain score	0.6 ± 0.7	1.3 ± 1	0.025

*p* values in italics represent statistical significance (*p* < 0.01)

### Complications

There were no major complications such as neurovascular injury, infection, or impaired wound healing. Surgery-related complication at 2-year follow-up included nonunion in 3 patients (11%), DRUJ subluxation in 3 patients (11%), implant migration in 4 patients (14%), and radiographic resorption of the ulnar styloid in 4 patients (14%). Radiographic nonunion was noted in 1 patient in group A (8%) and 2 in group B (13%). Residual DRUJ subluxation was noted in 3 patients; all were in group B (20%). Partial or complete radiographic resorption of the ulnar styloid was found in 1 patient in group A (8%) and 3 in group B (20%). Implant migration was noted in 1 patient in group A (8%), and 2 in group B (13%). Subsequent removal surgery due to implant irritation occurred in 13 patients (46%), with 4 in group A (31%) and 8 in group B (53%). A total of 11 patients (39%) with surgery-related complications included 5 (38%) in group A and 12 (80%) in group B, with a significant difference (*p* = 0.000) (Table 3).

**Table 3:** Surgical complications

Items	Group A	Group B	*p* value
Patients with complications	5 (38%)	12 (80%)	*0.000*
Bone related			
Nonunion	1 (8%)	2 (13%)	
DRUJ subluxation	0	3 (20%)	
Bone resorption	1 (8%)	3 (20%)	
Implant related			
Migration	1 (8%)	2 (13%)	
Removal	5 (38%)	8 (53%)	

*p* values in italics represent statistical significance (*p* < 0.01)

## Discussion

With comprehensive understanding of the ulnar structure of the wrist joint focused on the bony anatomy of the distal radioulnar joint and forearm rotation, the importance of the distal ulna in wrist biomechanics has been established [6]. The ulnar styloid process is currently recognized to play a critical role in functional capacity of the entire forearm to resume painless rotation stability [14]. However, the role of surgical fixation of the ulnar styloid fracture remains unclear. While several reports considered the effects of symptomatic instability or nonunion of ulnar styloid fractures [15, 16], the overwhelming benefits and potential implant complications with surgical procedures have not yet been justified [10]. Moreover, those studies only focused on the overall outcomes of DRF with concomitant ulnar styloid fixation instead of the surgical outcome of styloid fractures per se. To the best of our knowledge, our article is the first to compare the presentation and surgical outcome in early- and late-treated ulnar styloid fractures.

Ulnar styloid fractures have been anatomically classified as tip and base fractures [5]. The latter implies higher-energy trauma with a potential risk of distal radioulnar ligament tear and may serve as a marker for injury severity [17]. Articles favoring conservative treatment primarily emphasized successful surgical fixation in distal radius fractures, while the injury severity in ulnar styloid fractures per se and the resulting symptoms were generally underestimated and inadequately treated [12]. It is not our purpose to recommend that all ulnar styloid fractures need to be surgically treated. Instead, we aim to stress the importance of early detection of unstable ulnar styloid fractures, and both soft tissue and bony structures should be properly addressed to avoid late complications. In our series, a TFCC tear was found in 10 patients who underwent suture anchor repair during surgery. Concomitant soft tissue injuries around the DRUJ were commonly found with ulnar styloid fractures and could be overlooked with nonsurgical treatment. Our results suggested that early detection and surgical management achieved significantly better functional outcomes with fewer complications than with late intervention. Lower average pain scores were also found in the group with early fixation. While the difference was not significant, most patients showed no pain or only mild residual pain in both groups. This confirmed the effects of surgical intervention.

Ulnar styloid fractures have been traditionally discussed with distal radius fractures and generally treated conservatively [18]. Only a handful of articles have reported surgical management and outcomes of ulnar styloid fractures [1, 12, 19]. A current prospective study reported symptomatic nonunion that was successfully treated with plating osteosynthesis [12]. This study only enrolled patients with chronic injury occurring more than 6 months prior, and the sample size was small. Our study included 28 patients, and surgical outcomes and complications were compared between acute fracture and chronic injury. Since we used simple pins and wire to fix styloid fractures, and suture anchors for TFCC repair when necessary, the overall complication rate was higher than in a prior study using plating osteosynthesis [12]. There were more implant-related complications, especially in the chronic group. There were four patients with subsequent resorption of ulnar styloid; all were late-treated fractures (2.5, 4, 12, and 24 months after injuries). Yet no articles mentioned about ulnar styloid resorption. Possible detrimental factors including injury chronicity and suboptimal reduction-fixation could lead to postoperative bone resorption, which was commonly observed in proximal humerus fractures [20]. Better outcomes and lower complication rates were achieved in the group with early intervention, comparable to those in the prior report using plating osteosynthesis [12]. Nevertheless, the ideal implant for fixation of the ulnar styloid process is yet to be established. Potential surgical and implant-related complications still require surgeons to carefully select optimal candidates and treatment modalities.

Our study had all the limitations of a retrospective design by relying on medical records and operative notes for data collection. Despite being a case-comparison study, the surgical approach and fixation modality were empirically determined through the planning and experience of a single surgeon. The sample size was small in each group and lacked a control group. In addition, cases excluded due to insufficient follow-up may have had a substantial effect on outcome analysis.

## Conclusion

Ulnar styloid base fractures imply higher-energy trauma with potential ligamentous injury. Both osseous and soft tissue lesions need to be fully addressed. Early detection and surgical repair yielded better outcomes with lower complication rates. Both osseous and implant-related complications were common in late-treated fractures. Meticulous selection of surgical candidates and modalities is mandatory.

## Abbreviations

DRF: Distal radial fracture
DRUJ: Distal radioulnar joint
DSBUN: Dorsal sensory branch of ulnar nerve
QuickDASH: Quick Disabilities of the Arm, Shoulder, and Hand
TFCC: Triangular fibrocartilage complex
VAS: Visual analog scale
